# Association of circulating 25-hydroxyvitamin D with time in range and insulin secretion in type 2 diabetes

**DOI:** 10.3389/fendo.2025.1573963

**Published:** 2025-07-07

**Authors:** Wenwen Jiang, Ling Li, Wei Wang, Yue Liang, Xiaoyong Bai, Yixin Xu, Qingyu Guo, Lichao Ge, Jingjing Liang, Bin Lu, Jiaqing Shao

**Affiliations:** ^1^ Department of Endocrinology, Jinling Hospital, Nanjing Medical University, Nanjing, Jiangsu, China; ^2^ Department of Endocrinology, Chinese Navy No.971.Hospital, Qingdao, Shandong, China; ^3^ Department of Endocrinology, Jinling Hospital, Affiliated Hospital of Medical School, Nanjing University, Nanjing, Jiangsu, China; ^4^ Department of Endocrinology, Nanjing Jinling Hospital, Nanjing, Jiangsu, China; ^5^ Department of Endocrinology, Jinling Hospital, Southern Medical University, Nanjing, Jiangsu, China

**Keywords:** 25-hydroxyvitamin D, type 2 diabetes, time in range, homeostatic model assessment of insulin resistance, homeostatic model assessment of β cell function

## Abstract

**Background:**

To explore the association of circulating 25-hydroxyvitamin D [25(OH)D] with time in range and insulin secretion in type 2 diabetes.

**Methods:**

911 patients diagnosed with type 2 diabetes mellitus (T2DM) were included, and they underwent 3-day continuous glucose monitoring (CGM) and serum 25(OH)D measurements. The subjects were categorized into three groups based on the tertiles of their serum 25(OH)D levels: G1 (25(OH)D ≤ 21.22 ng/mL), G2 (21.22 ng/mL ≤ 25(OH)D < 26.43 ng/mL), and G3 (25(OH)D ≥ 26.43 ng/mL). TIR and glycemic variability (GV) parameters were evaluated with CGM. Insulin resistance was evaluated via the homeostatic model assessment of insulin resistance (HOMA-IR). The pancreatic β-cell function was determined using the homeostasis model assessment of β-cell function (HOMA-β). Among the 911 enrolled subjects, 582 individuals underwent a 100g standardized steamed bread meal test to comprehensively evaluate the pancreatic β-cell secretory function.

**Results:**

A higher TIR and time in tight range (TITR) were observed among individuals in the upper tertile for 25(OH)D, surpassing those in the middle and lower tertiles. The results of correlation analysis revealed that serum 25(OH)D levels were significantly positively correlated with TIR and TITR. Conversely, serum 25(OH)D levels were negatively associated with time above range, GV parameters, hemoglobin A1c (HbA1c), and HOMA-IR. However, no significant association was observed between serum 25(OH)D levels and HOMA-β. Additionally, the correlation between 25(OH)D and TIR (*r*=0.217, *P*<0.001) was slightly stronger than that between 25(OH)D and HbA1c (*r*=-0.130, *P*<0.001). Multiple stepwise linear regression analysis indicated that serum 25(OH)D levels were an independent influencing factor for TIR. Among individuals who underwent the steamed bread meal test, serum 25(OH)D exhibited a positive correlation with indicators of early-phase and overall pancreatic β-cell secretion capacity.

**Conclusions:**

This study demonstrated that in patients with T2DM, 25(OH)D levels exhibited positive correlations with TIR and glucose-stimulated insulin secretion parameters, while showing negative correlations with GV parameters. Lower serum 25(OH)D levels may adversely impact glucose homeostasis and pancreatic β-cell secretory function in T2DM patients.

## 1 Introduction

As a steroid hormone with pleiotropic functions, vitamin D exerts its physiological effects primarily through specific binding to the vitamin D receptor (VDR) (1). Beyond its classical role in calcium-phosphate metabolism, vitamin D actively modulates diverse pathophysiological processes, including immune regulation, inflammatory responses, and metabolic homeostasis (1). From another perspective, vitamin D potentially contributes significantly to the emergence and evolution of various diseases, among which type 2 diabetes is included. In recent years, the role of vitamin D in glucose metabolism has garnered increasing scientific interest. 1,25-Dihydroxyvitamin D (1,25(OH)_2_D) modulates glycemic regulation through multiple mechanisms. It binds to nuclear receptors to upregulate genes essential for insulin biosynthesis and secretion, thereby enhancing insulin production and release (2). Furthermore, via membrane receptor interactions, 1,25(OH)_2_D activates L-type voltage-gated calcium channels, elevates cytosolic calcium concentrations, triggers endoplasmic reticulum calcium release, and stimulates PKC/PKA signaling pathways, collectively potentiating insulin secretion from pancreatic β-cells (3). Additionally, vitamin D enhances insulin sensitivity by upregulating insulin receptor expression and functionality, promoting peripheral glucose uptake and utilization (4). These effects indicate that vitamin D plays a significant role in maintaining normal blood glucose levels. However, serum 25-hydroxyvitamin D [25(OH)D] levels in patients with type 2 diabetes mellitus (T2DM) are significantly lower than those in healthy controls (5, 6). Epidemiological studies have shown that the incidence of vitamin D deficiency in patients with T2DM can be as high as 64.2% (7).

Diabetes is a chronic disease with a high incidence, imposing a significant economic and social burden worldwide (8). Global estimates indicate that 529 million individuals were living with diabetes in 2021, with projections suggesting a rise to 1.31 billion by 2050. T2DM accounts for approximately 96% of all diabetes cases (9). Accurate glycemic control is vital in preventing diabetes and its complications, as both sustained hyperglycemia and significant glycemic variability exert detrimental effects on health (10–12). Hemoglobin A1c (HbA1c) is commonly regarded as the gold standard for assessing long-term glycemic control over a period of 2-3 months (12). Furthermore, several large-scale studies have confirmed a strong correlation between HbA1c levels and the risk of chronic complications of diabetes mellitus (12, 13). Nonetheless, HbA1c is considered to have some limitations, such as its failure to reflect hypoglycemic or hyperglycemic events, glycemic variability, and daily patterns of glycemia (14, 15). Consequently, relying exclusively on HbA1c may not accurately assess a patient’s glycemic control. Given these limitations of HbA1c, continuous glucose monitoring (CGM) technology has emerged with the advancement of medical technology. As the use of CGM becomes more widespread in clinical practice, which records blood glucose levels in real-time, an increasing number of CGM-derived metrics, including time above range (TAR), time in range (TIR), time below range (TBR), time in tight range (TITR) and glycemic variability (GV) metrics, have become prominent areas of research interest (15, 16). TIR refers to the percentage of time during a day that blood glucose levels fluctuate within the target range of 3.9-10 mmol/L. The newly proposed TITR further restricts the blood glucose range to 3.9-7.8 mmol/L, closer to healthy individuals’ physiological blood glucose conditions (17, 18). The American Diabetes Association advocates for TIR as a supplementary indicator of HbA1c for evaluating glycemic control (19). A significant correlation has been demonstrated between HbA1c and TIR (20). Multiple studies have now confirmed that TIR can serve as an independent predictor for the occurrence of long-term complications in diabetes and as an efficacy endpoint for clinical trials (21, 22).

Although prospective cohort studies have suggested that reduced serum 25(OH)D levels are significantly associated with an elevated risk of T2DM (23, 24), vitamin D supplementation trials have failed to establish causality (25, 26), suggesting the potential existence of unelucidated effector pathways or assessment bias. Current research on the vitamin D-diabetes relationship predominantly relies on static glycemic parameters such as HbA1c and fasting plasma glucose. These conventional metrics fail to capture the fluctuations in blood glucose levels, whereas CGM-derived dynamic parameters, including TIR and GV indices, enable more comprehensive evaluation of glucose homeostasis. Notably, the association patterns between vitamin D status and CGM-derived dynamic parameters, as well as their underlying mechanisms, remain unclarified. This knowledge gap may hinder the optimization of intervention strategies. To fill this gap and promote the development of intervention strategies for the prevention and treatment of diabetes, the aim of this study was to assess the correlations between serum 25(OH)D levels and TIR/GV parameters, in T2DM populations, while simultaneously investigating the associations of 25(OH)D levels with pancreatic β-cell function (including glucose-stimulated insulin secretion function) and insulin resistance. We hypothesized that serum 25(OH)D levels would exhibit significant positive correlations with TIR and inverse correlations with GV parameters. Furthermore, we proposed that serum 25(OH)D levels are linked to pancreatic β-cell secretory function, while showing no association with insulin resistance indices. This effect may be mediated through vitamin D’s capacity to enhance insulin secretion and attenuate glycemic fluctuations. Consequently, in vitamin D-related studies of patients with T2DM, TIR and GV parameters should be incorporated as critical endpoints alongside HbA1c, as they capture glycemic stability and fluctuation patterns that are not reflected by HbA1c. Moreover, investigations into β-cell function may provide mechanistic insights for subsequent research targeting molecular pathways underlying these observations.

## 2 Materials and methods

### 2.1 Research subjects

**Figure 1:**
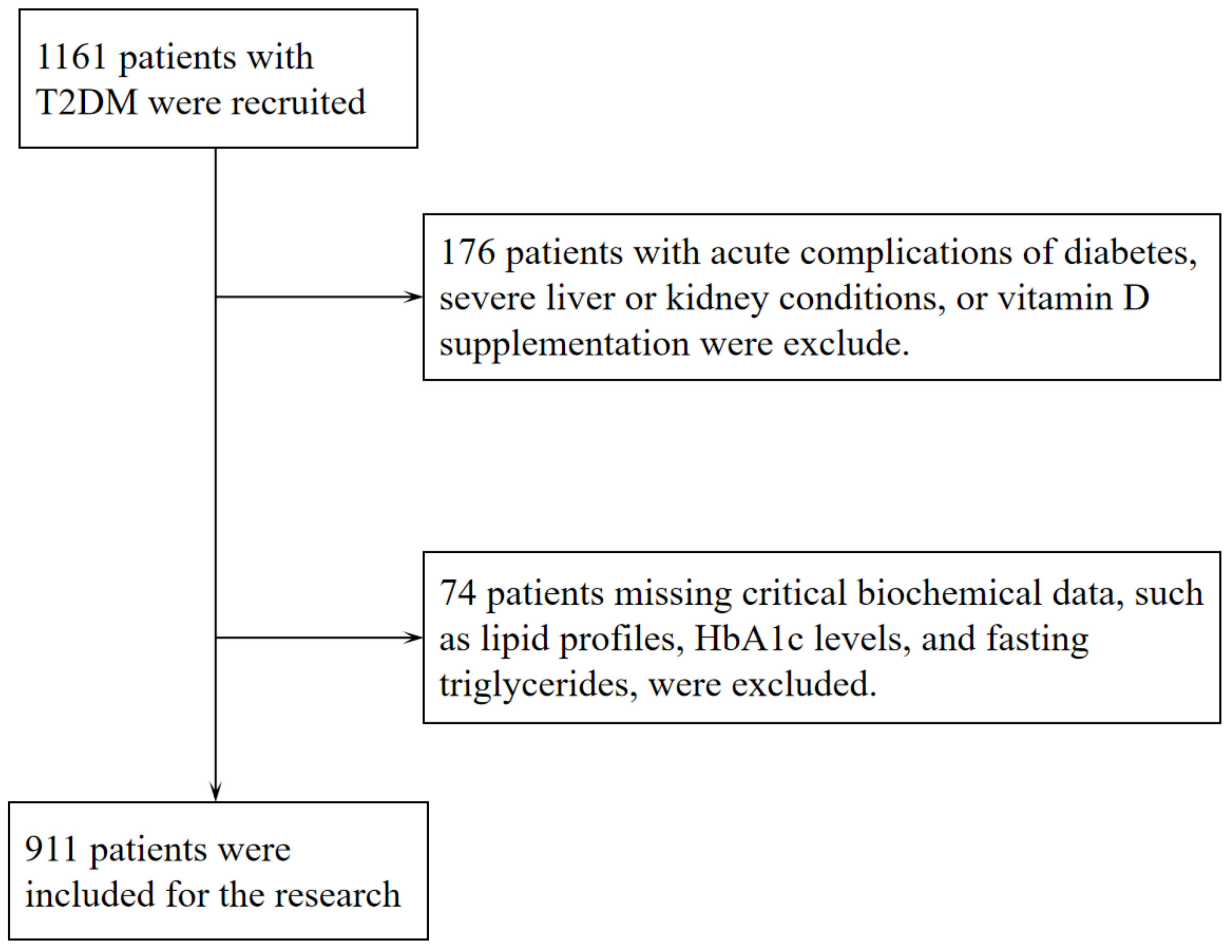
Flowchart of the participants included in the present study.

Following the diagnostic criteria defined by the World Health Organization in 1999 (27), 911 individuals (aged ≥18) with T2DM were enrolled in the Department of Endocrinology at Jinling Hospital of Nanjing Medical University from December 2016 to July 2022. As part of the standardized assessment protocol for hospitalized patients with T2DM at our institution, all participants underwent a 3-day CGM. All participants had been on stable hypoglycemic treatment within the 3 months before admission, and none had experienced episodes of diabetic ketoacidosis, hyperosmolar hyperglycemic state, or severe hypoglycemia. Exclusion criteria included (1) patients with a malignant tumor, acute stress (such as trauma, surgery, and severe infections), severe cardiovascular and cerebrovascular diseases, chronic liver disease, severe renal diseases and psychiatric disease; (2) patients with other endocrine diseases that can affect the skeleton or vitamin D (such as primary or secondary hyperparathyroidism and hyperthyroidism); (3) patients who have taken vitamin D, bisphosphonates, calcium supplements, or any other medications that may affect vitamin D synthesis and metabolism (such as rifampicin, phenytoin, isoniazid, glucocorticoids, etc.) in the past 6 months; (4) Patients with a history of fracture or orthopedic surgery in the past year; (5) some clinical baseline data were incomplete. Therefore, the final cohort of this study consisted of 911 participants. **Figure 1** depicts the precise workflow for the inclusion and exclusion of subjects in this study. Subsequently, according to the tertiles of serum 25(OH)D levels, patients were classified into 3 groups. The study protocol was approved by the Ethics Committee of Jinling Clinical Medical College, Nanjing Medical University, and conducted in accordance with the Declaration of Helsinki. All enrolled participants provided written informed consent prior to undergoing CGM during hospitalization. Among these, the 582 subjects who underwent the standardized 100g steamed bread meal test provided additional informed consent for this procedure before the test administration.

### 2.2 Clinical and biochemical information

General clinical information and physical examination results were obtained from the medical records. Sociodemographic characteristics and medical history consisted of gender, age, duration of disease, height, weight, diastolic blood pressure (DBP), systolic blood pressure (SBP), history of hypertensive disorder, alcohol consumption history, smoking history, complications of diabetes mellitus [including diabetic peripheral neuropathy (DPN), diabetic kidney disease (DKD) and diabetic retinopathy (DR)] and use of antihypertensive agents, antidiabetic therapy and lipid-lowering medications. SBP and DBP were measured twice by a specialist, and the mean values were recorded. Hypertension was diagnosed by an SBP of ≥140 mmHg and/or DBP of ≥90 mmHg or a history of taking antihypertensive medication. Smoking status was defined as non-smoking and smoking, where a non-smoker was considered a non-smoker or former smoker who had abstained from smoking for six months or more. Drinking status was defined as non-drinking and drinking, with non-drinking being considered as not having consumed alcohol or having previously consumed alcohol and having been abstinent for six months or more. Height and weight were measured and utilized to compute the body mass index (BMI). DPN was diagnosed based on symptoms reported during a neurological examination, including abnormalities in ankle reflexes, vibratory sensation, pressure sensation, pinprick sensation, and temperature sensation. DKD is characterized by urine albumin creatinine ratio (UACR) ≥30 mg/g and/or estimated glomerular filtration rate (eGFR) ≤60 ml/min/1.73 m². DR was characterized as the occurrence of non-proliferative or proliferative forms of the condition upon examination with a slit lamp and fundus examination or a history of laser photocoagulation treatment.

After an 8-hour fasting period in the hospital, peripheral venous blood samples were obtained from each patient the next morning to measure biochemical markers such as blood lipids, uric acid, creatinine, calcium, HbA1c, and fasting blood glucose levels. Urine samples were gathered from the patients to assess the levels of albumin and creatinine in urine, which were subsequently used to calculate the UACR. The eGFR was calculated using the CKD-EPI creatinine equation.

### 2.3 Measurement of serum 25(OH)D levels

The levels of serum 25(OH)D were measured using the electrochemiluminescence method (Elecsys, Roche Diagnostics). This competitive-binding assay (total assay time: 27 minutes) involved the following sequential steps: (1) pretreatment to release protein-bound 25(OH)D from vitamin D-binding protein (VDBP); (2) competitive binding between sample 25(OH)D and ruthenium-labeled VDBP; (3) solid-phase capture of biotinylated 25(OH)D with streptavidin-coated magnetic particles, retaining unoccupied ruthenium-labeled VDBP; (4) electrochemical excitation to generate luminescent signals inversely proportional to 25(OH)D concentration; and (5) automated calculation via calibration curves. The assay demonstrated an analytical measurement range of 3.00–70.0 ng/mL, with 100% cross-reactivity to 25(OH)D_3_ and 92% to 25(OH)D_2_. Intra-assay coefficients of variation were 1.7% [25(OH)D mean concentration: 67.0 ng/mL] and 7.8% [25(OH)D mean concentration: 6.76 ng/mL], while inter-assay coefficients of variation were 2.2% [25(OH)D mean concentration: 67.0 ng/mL] and 13.1% [25(OH)D mean concentration: 6.76 ng/mL]. The limit of detection was 3.00 ng/mL, with a lower limit of quantification at 5.00 ng/mL. Method validation against LC-MS/MS yielded the regression equation y = 1.09x - 0.510 (*r* = 0.894).

### 2.4 Continuous glucose monitoring

During the study period, all participants wore a CGM system (Meiqi, China) device on either the right or left upper arm, automatically recording subcutaneous interstitial glucose every 3 minutes for 72 hours. Capillary glucose measurements were entered more than four times a day by a specialized nurse to calibrate CGM system. Participants were prohibited from strenuous exercise and showering during the glucose monitoring period. Based on the raw glycemic data from this system, Easy GV version 9.0R2 served as the tool for computing the CGM-derived key metrics (including TIR, TBR, TAR, coefficient of variation (CV) of blood glucose, standard deviation (SD), mean amplitude of glycemic excursion (MAGE), and mean blood glucose (MBG) to assess glycemic control.

### 2.5 Evaluation of pancreatic β-cell function and insulin resistance

Fasting blood glucose and fasting insulin levels were assessed in all participants to determine insulin resistance and pancreatic β-cell function. The homeostasis model assessment of β cell function (HOMA-β) was calculated by the formula 20*fasting insulin (mIU/L)/(fasting serum glucose(mmol/L)-3.5), which reflected the function of basal insulin secretion. The homeostatic model assessment of insulin resistance (HOMA-IR) was calculated by the formula fasting serum glucose(mmol/L)*fasting insulin (mIU/L)/22.5. Additionally, to further investigate the relationship between serum 25(OH)D levels and pancreatic β-cell function, a portion of the participants engaged in a 100g standard steamed bun meal test, which is used as a substitute for the traditional Oral Glucose Tolerance Test (OGTT) (28). The 100g standard steamed bun meal induced changes in insulin and C-peptide levels that were comparable to those observed following a 75g glucose intake, while also reducing the gastrointestinal discomfort associated with ingesting a large amount of glucose orally. The participants fasted for at least 8 hours before the test. An insulin-C-peptide release test was conducted concurrently within the first 3 hours after the steamed bun meal test. Serum glucose, serum C-peptide, and serum insulin concentrations were evaluated at various time points following meals. The formula for the Insulinogenic Index (IGI) was IGI = ΔInsulin (0. 5–0 h)/ΔGlucose (0.5–0 h). The area under the C-peptide curve (AUC_Cp_) for 30 minutes and 180 minutes was determined using the trapezoidal method. IGI and AUC_Cp30_ both indicate early-phase insulin secretion function, whereas AUC_Cp180_ represents the overall β-cell secretion capacity. During the testing interval, all antidiabetic therapies were discontinued, and the administration of basal insulin was suspended the previous night.

### 2.6 Statistical analysis

Research data was analyzed with Statistical Product and Service Solutions 25.0 (SPSS 25.0) and Graph Pad Prism software. Continuous quantitative data conformed to normal distribution by the Shapiro–Wilk test was described as mean ± standard deviation, those conformed to skewed distribution were described as median (interquartile). Categorical data were described as counts (percentages). Comparisons between groups were achieved by the one-way ANOVA test for normally distributed data, the Kruskal–Wallis H-test for non-normally distributed data, and the chi-squared test for categorical data. Spearman rank analysis was adopted to initially explore the correlation of 25(OH)D with TIR, GV parameters, HOMA-IR, and pancreatic β-cell function indicators. Fisher’s r-to-z transformation test was performed to compare differences between correlation coefficients, aiming to validate whether the associations of 25(OH)D with TIR and HbA1c exhibited statistically significant differences. Linear stepwise multiple regression analysis was applied to determine the influence factors of TIR, TITR, TAR, HbA1c, HOMA-IR, and HOMA-β. *P* < 0.05 (Two-tailed) was considered statistically significant.

## 3 Results

### 3.1 Comparison of baseline characteristics among different groups

911 patients with T2DM were included in this observational research. All participants had a median (lower and upper quartiles) serum 25(OH)D levels of 23.80(19.71,28.31) ng/mL, age of 56.00 (48.00, 65.00) years, diabetes duration of 7.00 (2.00, 13.00) years, and HbA1c level of 8.50 (7.20, 10.00) %. The subjects were categorized into three groups based on the tertiles of their serum 25(OH)D levels: G1 (25(OH)D ≤ 21.22 ng/mL), G2 (21.22 ng/mL ≤ 25(OH)D < 26.43 ng/mL), and G3 (25(OH)D ≥ 26.43 ng/mL). **Table 1** provides a comprehensive overview of the participants’ baseline clinical features at the study’s outset. Group G1, in contrast to G3, had lower HDL levels, higher levels of TC and HbA1c, and a higher prevalence of DR. G1 also had higher levels of UACR and FBG, as well as a higher prevalence of DKD, when compared to the other two groups. On the other hand, G3 had a lower prevalence of DPN and lower levels of TG compared to G1 and G2. No statistically significant differences were observed among all groups in terms of gender, age, diabetes duration, smoking, alcohol use, hypertension, DBP, BMI, calcium, ALT, AST, LDL-C, Scr, SUA, eGFR, HOMA-β, HOMA-IR, fasting insulin, and fasting C-peptide (FCp). Regarding medication usage, apart from insulin, DPP-4 inhibitors and SGLT-2 inhibitors, there were no statistically significant differences among the three groups in the use of other common oral hypoglycemic agents, antihypertensive medications, aspirin, and statins for lipid-lowering.

**Table 1:** The comparison of baseline characteristics by tertiles (G1-G3) of 25(OH)D levels.

Variables	G1	G2	G3	*P*
Number	304	304	303	
Male (%)	190(62.50)	192(63.20)	212(70.00)	0.102
Age (years)	55(46,65)	56(49,65)	57(49,67)	0.118
Duration (years)	7.5(2,14)	7(2,13)	7(3,12)	0.974
Smoking n (%)	67(22.00)	73(24.00)	71(23.40)	0.839
Drinking n (%)	47(15.50)	42(13.80)	53(17.50)	0.457
Hypertension n (%)	171(56.30)	162(53.30)	156(51.50)	0.493
SBP (mmHg)	132(124,146)	130(120,142) ^*^	130(120,141)	0.044
DBP (mmHg)	80(74,88)	79.5(74,86.75)	79(72,87)	0.153
BMI (kg/m^2^)	25.33(23.03,27.74)	25.09(22.61,27.34)	25.10(22.58,26.89)	0.307
Ca (mmol/L)	2.19(2.12,2.26)	2.20(2.14,2.26)	2.19(2.12,2.27)	0.375
ALT (U/L)	18(13,27)	19(13,26)	19(14,29)	0.550
AST (U/L)	17(13,21)	16(13.25,20.75)	17(14,23)	0.197
TC (mmol/L)	4.61(3.92,5.35)	4.41(3.69,5.20)	4.31(3.61,5.04) ^#^	0.009
TG (mmol/L)	1.79(1.15,2.63)	1.59(1.09,2.53)	1.33(0.95,2.01) ^*#^	<0.001
HDL-C (mmol/L)	1.01(0.88,1.22)	1.05(0.88,1.20)	1.08(0.93,1.27) ^*^	0.017
LDL-C (mmol/L)	2.68(2.17,3.32)	2.66(2.00,3.34)	2.64(2.01,3.32)	0.796
Scr (mmol/L)	58(46.2,72.13)	57(46,68.58)	58(50.2,69)	0.27
SUA (mmol/L)	318.5(260.25,393)	309.5(259,376.75)	319(267,376)	0.365
UACR (mg/g)	15.89(6.80,123.57)	12.04(6.41,33.80) ^*^	10.20(4.99,28.67) ^*^	<0.001
HbA1c (%)	8.90(7.60,10.30)	8.50(7.23,10.08)	8.10(6.90,9.70) ^*^	0.001
eGFR (ml/min per 1.73 m^2^)	105.49(91.52,118.80)	105.81(95.58,114.88)	103.91(93.41,113.65)	0.458
HOMA-IR	2.11(1.04,4.44)	2.31(1.07,4.70)	1.74(0.78,4.13)	0.093
HOMA-β	27.87(13.79,79.32)	40.42(15.86,89.26)	34.46(15.81,98.18)	0.066
FINS(μU/ml)	5.45(2.70,12.58)	6.98(3.22,14.13)	5.84(2.10,12.80)	0.132
FCp (pmol/L)	1.32(0.80,2.10)	1.41(0.81,1.99)	1.40(0.77,2.01)	0.986
FBG (mmol/L)	7.70(6.40,9.40)	7.10(5.90,9.00) ^*^	6.90(5.70,8.40) ^*^	<0.001
DR n (%)	98(32.20)	81a, (26.60)	68(22.40) ^*^	0.025
DKD n (%)	98(32.20)	42(13.80) ^*^	44(14.50) ^*^	<0.001
DPN n (%)	110(36.20)	93(30.60)	69(22.80) ^*#^	0.001
Anti-diabetic treatment n (%)				
Insulin	211(69.4)	191(62.8)	166(54.8)*	0.001
glucagon like peptide-1	48(15.8)	55(18.1)	51(16.8)	0.750
Metformin	210(69.10)	217(71.40)	216(71.30)	0.78
Sulfonylureas	15(4.90)	20(6.60)	27(8.90)	0.148
Glinides	28(9.20)	32(10.50)	27(8.90)	0.771
α-glucosidase inhibitors	110(36.20)	109(35.90)	121(39.90)	0.514
Thiazolidinediones	18(5.90)	13(4.30)	16(5.30)	0.652
DPP-4 inhibitors	87 (28.60)	105(34.50)	66(21.80) ^#^	0.002
SGLT-2 inhibitors	11(3.60)	1(0.30) ^*^	9(3.00) ^#^	0.017
Anti-hypertensive treatment n (%)				
RAAS inhibitors	131 (43.1)	111 (36.5)	112 (37)	0.178
Calcium channel blockers	102 (33.6)	90 (29.6)	103 (34)	0.445
b-blockers	38 (12.5)	32 (10.5)	28 (9.2)	0.426
Diuretics	26 (8.6)	29 (9.5)	26 (8.6)	0.888
Aspirin therapy n (%)	68 (22.4)	55 (18.1)	63 (20.8)	0.417
Statin therapy n (%)	118 (38.8)	101 (33.2)	126 (41.6)	0.096

Continuous variables are presented as means ± SD and medians (lower and upper quartiles), and categorical variables are expressed as numbers (percentages).

SBP, systolic blood pressure; DBP, diastolic blood pressure; BMI, body mass index; ALT, alanine aminotransferase; AST, aspartate aminotransferase; TC, total cholesterol; TG, triglyceride; HDL-C, high-density lipoprotein cholesterol; LDL-C, low-density lipoprotein cholesterol; Scr, serum creatinine; SUA, serum uric acid; UACR, urine albumin creatinine ratio; HbA1c, Hemoglobin A1c; eGFR, estimated glomerular filtration rate; HOMA-IR, homeostatic model assessment of insulin resistance; HOMA-β, homeostasis model assessment of β cell function; FINS, Fasting insulin; FCp, Fasting C-peptide; FBG, fasting blood glucose; DR, diabetic retinopathy; DKD, diabetic kidney disease; DPN, diabetic peripheral neuropathy; DPP-4 inhibitors, Dipeptidyl Peptidase-4 inhibitors; SGLT-2 inhibitors, sodium glucose cotransporter 2 inhibitors; RAAS inhibitors, renin-angiotensin-aldosterone system inhibitors.

G1 (25 (OH)D<21.22 ng/mL), G2 (21.22 ng/mL ≤ 25 (OH)D <26.43 ng/mL), G3 (25 (OH)D≥26.43 ng/mL).

^*^ Significant difference with group 1 (*P* < 0.05).

^#^ Significant difference with group 2 (*P* < 0.05).

### 3.2 Comparison of CGM-derived metrics among different groups

The other two groups showed lower TITR and TIR compared to Group G3, while Group G3 had lower levels of TAR and SD. MBG value decreased with the increasing tertiles of serum 25(OH)D. No significant discrepancies were observed in TBR, CV, and MAGE among the three groups. Overall, Group G3 exhibited the most stable glycemic control. **Table 2** provides a comprehensive description of the participants’ CGM-derived metrics.

**Table 2:** The comparison of CGM-derived metrics by tertiles (G1-G3) of 25(OH)D levels.

Variables	G1	G2	G3	*P*
Number	304	304	303	
TBR (%)	0(0,0)	0(0,0)	0(0,0)	0.489
TITR (%)	25.21(7.03,46.82)	32.60(9.74,56.67)	35.67(19.17,63.54) ^*#^	<0.001
TIR (%)	62.14(37.85,80.12)	69.97(43.82,84.10)	75.60(57.56,88.80) ^*#^	<0.001
TAR (%)	36.76(19.20,62.16)	29.59(14.84,56.19)	24.22(10.62,41.66) ^*#^	<0.001
MBG (mmol/L)	9.68(8.33,11.27)	9.22(8.13,10.68) ^*^	8.75(7.68,9.87) ^*#^	<0.001
SD	2.40(1.90,3.06)	2.26(1.75,2.98)	2.09(1.61,2.76) ^*#^	<0.001
CV	0.25(0.20,0.30)	0.25(0.19,0.30)	0.23(0.20,0.29)	0.265
MAGE (mmol/L)	4.31(3.34,5.58)	4.26(3.33,5.26)	4.07(3.11,5.17)	0.131

Continuous variables are presented as means ± SD and medians (lower and upper quartiles), and categorical variables are expressed as numbers (percentages).

TBR, time above range; TITR, time in tight range; TIR, time in range; TAR, time below range; MBG: mean blood glucose; SD, standard deviation; CV, coefficient of variation; MAGE, mean amplitude of glycemic excursions.

G1 (25 (OH)D<21.22 ng/mL), G2 (21.22 ng/mL ≤ 25 (OH)D <26.43 ng/mL), G3 (25 (OH)D≥26.43 ng/mL).

^*^ Significant difference with group 1 (*P* < 0.05).

^#^ Significant difference with group 2 (*P* < 0.05).

### 3.3 The correlation of TIR and GV parameters with serum 25(OH)D

**Figure 2:**
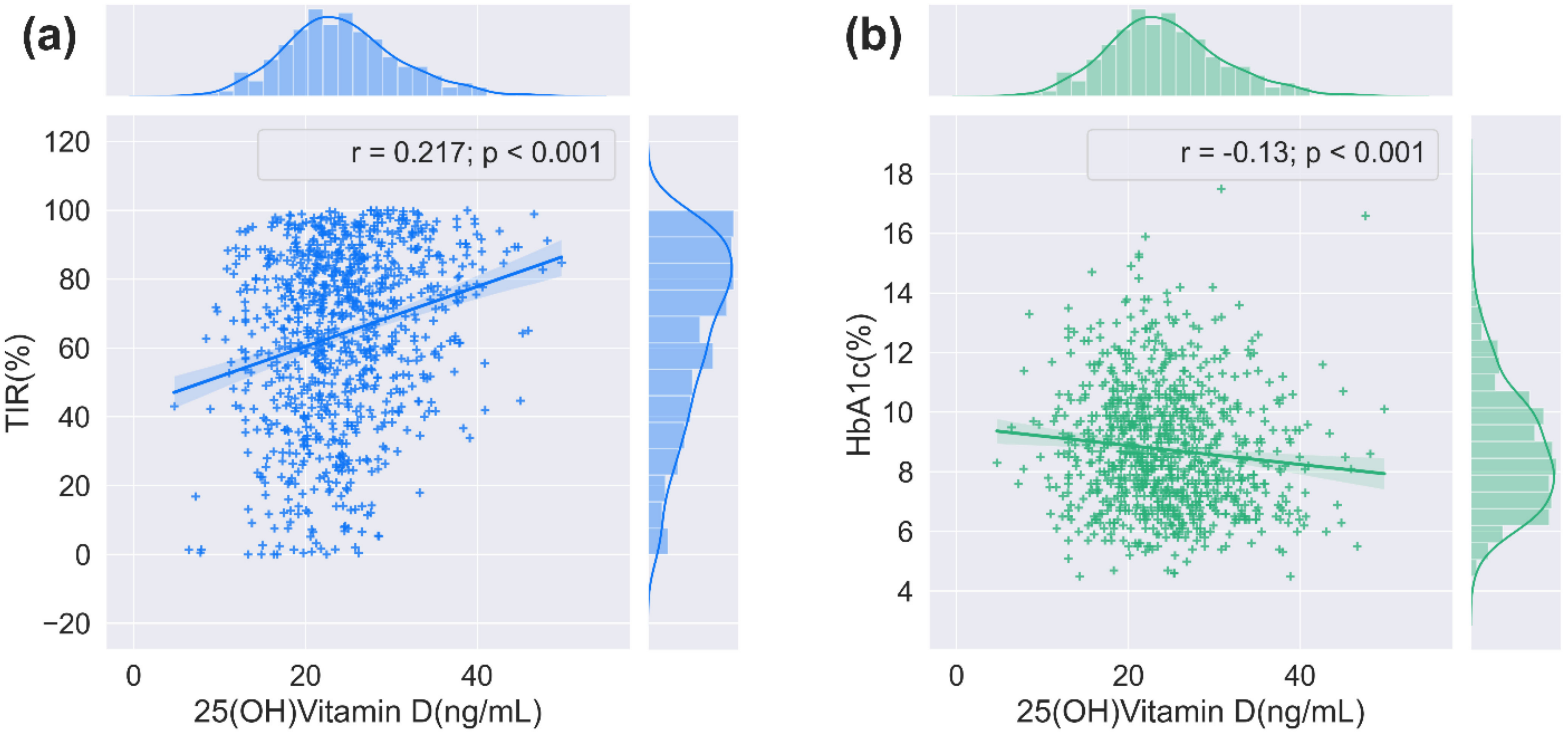
The correlation between 25(OH)D and TIR/HbA1c. **(a)** The relationship between serum 25(OH)D and TIR (n = 911, 2-tailed Spearmen correlation). **(b)** The relationship between serum 25(OH)D and HbA1c (n = 911, 2-tailed Spearmen correlation).

The relationship between serum 25(OH)D and glycemic control indexes was evaluated using Spearman’s correlation analysis, which indicated significant positive associations between serum 25(OH)D and TIR, and TITR (*r* = 0.217, and 0.192, respectively, *P* < 0.001). Conversely, negative correlations were identified with TAR, SD, CV, MBG, MAGE, HbA1c, and HOMA-IR (*r* = -0.216, -0.164, -0.067, -0.212, -0.077, -0.130, and -0.069, respectively, *P* < 0.05). No significant correlation was noted between serum 25(OH)D and TBR or HOMA-β. Subsequent application of Fisher’s r-to-z transformation test to compare the correlation between 25(OH)D and TIR (*r* = 0.217) versus that of 25(OH)D and HbA1c (*r* = -0.130) revealed a statistically significant discrepancy (*z* = 7.47, *P* < 0.001) as shown in **Figure 2**. **Table 3** describes the correlation between 25(OH)D and CGM-derived metrics.

**Table 3:** The correlation of TIR and GV parameters with 25(OH)D by Spearman’s analysis.

Variables	*r*	*P*
TBR (%)	0.030	0.371
TITR (%)	0.192	<0.001
TIR (%)	0.217	<0.001
TAR (%)	-0.216	<0.001
MBG (mmol/L)	-0.212	<0.001
SD	-0.164	<0.001
CV	-0.067	0.042
MAGE (mmol/L)	-0.077	0.019
HbA1c (%)	-0.130	<0.001
HOMA-IR	-0.069	0.039
HOMA-β	0.060	0.070

TBR, time above range; TITR, time in tight range; TIR, time in range; TAR, time below range; MBG: mean blood glucose; SD, standard deviation; CV, coefficient of variation; MAGE, mean amplitude of glycemic excursions; HbA1c, Hemoglobin A1c; HOMA-IR, homeostatic model assessment of insulin resistance; HOMA-β, homeostasis model assessment of β cell function.

### 3.4 Multiple stepwise regression analysis of the factors influencing TIR, TITR, TAR, HbA1c, HOMA-IR, and HOMA-β

Multivariate stepwise regression analysis was employed to assess the factors influencing TIR, TITR, TAR, HbA1c, HOMA-IR, and HOMA-β. The results showed that serum 25(OH)D is an independent factor for TIR, TITR, TAR, and HbA1c but not for HOMA-IR and HOMA-β. The detailed results are presented in **Tables 4**–**7** and **Supplementary Tables 1**, **2**.

**Table 4:** Multiple stepwise regression analysis of influencing factors of TIR.

Variables	β	t	*P*	95%CI
(Constant)	79.108	9.293	<0.001	62.400 to 95.815
HbA1c	-4.950	-13.750	<0.001	-5.657 to -4.244
TG	-1.667	-5.982	<0.001	-2.214 to -1.120
Duration	-0.669	-6.519	<0.001	-0.870 to -0.468
25(OH)D	0.491	4.536	<0.001	0.278 to 0.703
UA	0.023	2.826	0.005	0.007 to 0.038
HDL-C	8.429	2.901	0.004	2.726 to 14.133
UACR	-0.002	-2.320	0.021	-0.004 to 0.000
ALT	-0.068	-2.401	0.017	-0.124 to -0.012
BMI	0.455	2.110	0.035	0.032 to 0.878

dependent variable: TIR

**Table 5:** Multiple stepwise regression analysis of influencing factors of HbA1c.

Variables	β	t	*P*	95%CI
(Constant)	10.924	20.986	<0.001	9.903 to 11.946
LDL-C	0.495	6.727	<0.001	0.351 to 0.64
UA	-0.004	-6.159	<0.001	-0.006 to -0.003
HDL-C	-0.772	-2.855	0.004	-1.303 to -0.241
TG	0.081	3.166	0.002	0.031 to 0.131
Duration	-0.019	-2.044	0.041	-0.037 to -0.001
25(OH)D	-0.03	-3.035	0.002	-0.049 to -0.01
Gender	-0.375	-2.571	0.01	-0.661 to -0.089

dependent variable: HbA1c

**Table 6:** Multiple stepwise regression analysis of influencing factors of HOMA-IR.

Variables	β	t	*P*	95%CI
(Constant)	7.237	5.797	<0.001	4.787 to 9.687
Duration	0.212	5.102	<0.001	0.131 to 0.294
HDL-C	-3.965	-3.556	<0.001	-6.154 to -1.777

dependent variable: HOMA-IR

**Table 7:** Multiple stepwise regression analysis of influencing factors of HOMA-β.

Variables	β	t	*P*	95%CI
(Constant)	235.417	3.309	0.001	95.786 to 375.048
UACR	0.107	4.106	<0.001	0.056 to 0.158
Duration	4.92	1.856	0.064	-0.283 to 10.123
TG	-18.222	-2.581	0.01	-32.079 to -4.366
LDL-C	-43.232	-2.056	0.04	-84.503 to -1.961

dependent variable: HOMA-β

### 3.5 The correlation of pancreatic β-cell function with serum 25(OH)D in certain population segments

In our research, 582 participants underwent a 100g standard steamed bread meal test to further evaluate pancreatic β-cell secretory function. The clinical baseline characteristics of these participants are presented in **Supplementary Table 3**. Spearman correlation analysis revealed a substantial positive association between serum 25(OH)D concentrations and various measures, including INS1h, INS2h, Cp0.5h, Cp1h, Cp2h, Cp3h, IGI, the area under the C-peptide curve within 30 minutes (AUC_Cp30_), and the area under the C-peptide curve over three hours (AUC_Cp180_), with correlation coefficients of *r*=0.097, 0.124, 0.111, 0.139, 0.171, 0.134, 0.122, 0.093, and 0.147, respectively. However, no correlation was found between serum 25(OH)D and HOMA-β. The specific details are presented in **Table 8**.

**Table 8:** The correlation of pancreatic β-cell function with 25(OH)D in certain population segments by Spearman’s analysis.

Variables	*r*	*P*
INS0h	-0.001	0.987
INS0.5h	0.059	0.158
INS1h	0.097	0.02
INS2h	0.124	0.003
INS3h	0.063	0.128
Cp0h	0.06	0.149
Cp0.5h	0.111	0.007
Cp1h	0.139	0.001
Cp2h	0.171	<0.001
Cp3h	0.134	0.001
IGI	0.122	0.003
AUC_Cp30_	0.093	0.024
AUC_Cp180_	0.147	<0.001
HOMA-β	0.051	0.222

INS, insulin; Cp: C-peptide; IGI, Insulinogenic index; AUC_Cp_, the area under the curve of C-peptide; HOMA-β, homeostasis model assessment of β-cell function.

## 4 Discussion

In this study, we investigated the cross-sectional relationships between serum 25(OH)D levels and TIR as well as insulin secretory function in a substantial cohort. Our findings demonstrated that serum 25(OH)D levels were positively correlated with TIR and glucose-stimulated insulin secretion indices, while exhibiting negative correlations with GV parameters.

In recent years, the non-skeletal impacts of vitamin D have attracted wide attention, with increasing evidence suggesting that vitamin D can participate in glucose metabolism through various mechanisms (29–32). Previous studies have predominantly focused on traditional glycemic metrics such as HbA1c and spot blood glucose measurements. In contrast, this study innovatively introduces a novel CGM-derived parameter system, including TIR and TITR. CGM systems precisely capture 24-hour glycemic profiles in individuals, comprehensively reflecting daytime glucose fluctuations, hyperglycemic/hypoglycemic events, and GV characteristics, thereby effectively addressing the limitations of conventional glucose monitoring methods (15). TIR not only provides real-time insights into short-term glycemic control. Still, it is also instrumental in forecasting the onset of diabetes-related complications and evaluating the outcomes of clinical trials (17, 21, 22). TITR refers to the period in which blood glucose concentrations are kept within a narrower band (3.9–7.8 mmol/L) than that for TIR (3.9–10.0 mmol/L). It better reflects the blood glucose patterns of the normal population, thereby enabling more precise glycemic management while minimizing significant glucose fluctuations (18). The latest 2025 International Conference on Advanced Technologies and Treatments for Diabetes has proposed TITR as a complementary assessment metric to TIR for glycemic monitoring (33). Additionally, HbA1c is closely correlated with both TIR and TITR (18, 20). Our study indicated a notable and positive correlation between 25(OH)D levels and both TIR and TITR, while a negative correlation was observed with TAR. The correlation coefficients of serum 25(OH)D with TIR and TITR are nearly equivalent. Other CGM-derived metrics, including MBG, SD, CV, and MAGE, exhibited a certain degree of association with serum 25(OH)D levels. Furthermore, Fisher’s r-to-z transformation test confirmed that the correlation between 25(OH)D and TIR was significantly stronger than the correlation between 25(OH)D and HbA1c (*z*=7.47, *P*<0.001). This finding suggests that vitamin D may optimize glycemic control by ameliorating short-term glycemic fluctuations rather than solely modulating long-term average glucose levels (as reflected by HbA1c).

The most typical characteristics of type 2 diabetes are insulin resistance accompanied by pancreatic β-cell dysfunction. In this study, we used HOMA-IR to assess the degree of insulin resistance. We observed a weak correlation between 25(OH)D levels and HOMA-IR (*r*=-0.069, *P*=0.039). After adjusting for confounding factors, we found that vitamin D levels were not an independent influencing factor for HOMA-IR. This is consistent with the findings of Liu et al. (34), whereas Geng et al. observed a certain correlation between serum 25(OH)D and HOMA-IR in their study (35). This discrepancy may be attributed to differences in the ethnic backgrounds of the study populations, as Geng et al. primarily focused on people from the United States. Additionally, we used HOMA-β to evaluate the insulin secretion function of the participants. We found no significant correlation between serum 25(OH)D levels and HOMA-β. This result echoes a recent study, which similarly observed no significant correlation between 25(OH)D and HOMA-β. However, they found 1,25(OH)2D to be positively correlated with HOMA-β. Notably, the study had a relatively small sample size. To comprehensively investigate the association between vitamin D and pancreatic β-cell function, a further analysis was conducted on the population that participated in the 100g standard steamed bread meal test within this study. Our findings reveal a positive association between serum 25(OH)D and INS1h, INS2h, Cp0.5h, Cp1h, Cp2h, Cp3h, INS1h, INS2h, IGI, AUC_Cp30_, and AUC_Cp180_, while no correlation was found with HOMA-β. These observations are consistent with the findings reported by Liu and colleagues (34). These findings suggest that vitamin D may predominantly influence glucose-stimulated insulin secretion in pancreatic β-cells of patients with T2DM, with no significant effect on basal insulin secretion. Recent *in vitro* experiments demonstrated that 1,25-dihydroxyvitamin D [1,25(OH)_2_D] intervention significantly enhanced glucose-stimulated insulin secretion in INS1E cells while leaving basal secretion unaffected (29). This phenomenon may arise from β-cell-specific expression of the VDR and 1α-hydroxylase system, which provides a molecular basis for local vitamin D activation and potentially underlies its selective association with glucose-stimulated insulin secretory function (30, 31). Upon binding to VDR, 1,25(OH)_2_D upregulates insulin biosynthesis/secretion-related genes and calcium channel proteins, thereby potentiating insulin production and release (2, 32). In addition, 1,25(OH)_2_D can also regulate the activity of L-type voltage-gated calcium channels (L-VDCC), K^+^-ATP channels, K^+^-Ca^2+^ channels, and Kv channels by binding to membrane receptors, rapidly increasing cytoplasmic calcium concentration, triggering endoplasmic reticulum calcium release, and activating the PKC/PKA signaling pathway, thereby further enhancing secretion (3). Vitamin D has also been demonstrated to significantly reduce the ratio of fasting proinsulin to C-peptide in individuals with newly diagnosed type 1 diabetes mellitus, suggesting a protective effect on β-cell function (36). These synergistic mechanisms likely contribute to postprandial β-cell functional enhancement. Although this study reveals an association between vitamin D status and postprandial β-cell function, suggesting that vitamin D may be a potential target for improving postprandial glycemic fluctuations in patients with T2DM, the cross-sectional design limitations preclude establishing a causal relationship. Further validation through longitudinal cohort studies is required.

Recent studies have conclusively demonstrated that serum 25(OH)D is involved in the regulation of glucose metabolism through multiple pathways. A meta-analysis by Zhang Y. et al. demonstrated that vitamin D supplementation effectively reduces the risk of progression from prediabetes to diabetes mellitus, while enhancing the probability of restoring normal glycemic parameters in this population (37). Notably, in 2024, the updated clinical guidelines from the Endocrine Society of the United States for the first time incorporated recommendations on vitamin D’s non-skeletal effects, explicitly endorsing evidence-based preventive vitamin D supplementation regimens for individuals with prediabetes (38). Nevertheless, in the context of T2DM management, controversies persist regarding the evidence for vitamin D and its analogs in regulating glycemic homeostasis and preventing complications. A recent meta-analysis revealed that vitamin D intervention improves FBG, HbA1c, insulin levels, and HOMA-IR in patients with T2DM, particularly among those with baseline vitamin D deficiency (39). In contrast, another study demonstrated no significant improvement in HbA1c, FBG, or HOMA-IR even in T2DM patients with baseline vitamin D deficiency. However, this study noted a significant FBG improvement in the subgroup with suboptimal baseline glycemic control (40). These studies predominantly employed HbA1c as a surrogate indicator for patients’ glycemic control rather than TIR. Given that many interventional studies have short durations of intervention and participants come from diverse racial backgrounds, relying solely on HbA1c as the primary outcome measure in clinical trials may not provide a comprehensive assessment. Additionally, there is a lack of research that uses TIR as a clinical endpoint in vitamin D intervention experiments despite TIR being validated as an effective endpoint in numerous large-scale studies. Our study further confirmed that the correlation between TIR and vitamin D is not inferior to that of HbA1c. Therefore, we recommend that future prospective intervention studies integrate TIR with conventional glycemic parameters to achieve a multidimensional assessment of glycemic control quality. This integrative approach will facilitate data-driven optimization of personalized vitamin D supplementation protocols, thereby enhancing precision in clinical decision-making pathways.

Some limitations of this study should be noted. First of all, this is a retrospective observational study providing less evidence than RCTs, cohort studies, or case-control studies. We could not investigate the causality of the temporal correlation between vitamin D and TIR since it adopts a cross-sectional design. Secondly, this study is a single-center study conducted in a central city in China, based on which our findings may not be extendable to all diabetic patients of other ethnicities. Thirdly, all participants in this study underwent only 3-day CGM rather than the internationally recommended 14-day protocol (41). While prior evidence suggests short-term 3-day CGM can provide preliminary assessment of glycemic control (42, 43), cautious interpretation of our findings is warranted. Nevertheless, these preliminary data establish a foundation for future longitudinal investigations. Finally, future studies should take into account the dynamic nature of serum 25(OH)D levels, which can be influenced by various factors, including outdoor activities, diet, clothing choices, sunscreen application, and exercise. Therefore, it would be beneficial to include both baseline and periodic follow-up assessments of serum 25(OH)D levels to capture their fluctuations over time. Overall, our results call for more large-scale multicenter prospective studies to corroborate our findings, as well as basic experiments to explore the physiological mechanisms between vitamin D levels and glycemic control.

## 5 Conclusions

In conclusion, this study revealed that serum 25(OH)D levels in patients with T2DM were positively correlated with TIR, with this association being significantly stronger than the relationship between 25(OH)D and HbA1c. Moreover, serum 25(OH)D levels exhibited inverse correlations with GV parameters and positive correlations with glucose-stimulated insulin secretion indices. Low 25(OH)D level may adversely affect glucose homeostasis and β-cell insulin secretory function in patients with T2DM. These findings provide novel evidence supporting the potential role of vitamin D supplementation in diabetes management. Future interventional studies should incorporate TIR as an endpoint alongside HbA1c to comprehensively evaluate the therapeutic effects of vitamin D on glycemic regulation.

## Data Availability

The datasets presented in this article are not readily available because the data generated in the current study are available from the corresponding author on reasonable request. However, the availability of these data is limited and could be used under the permission of the current stud, and thus are not available to public. Requests to access the datasets should be directed to Wenwen Jian, jiangwenwenpink@163.com.
