# Supplementary material for: Association of circulating 25-hydroxyvitamin D with time in range and insulin secretion in type 2 diabetes
Source: Front Endocrinol (Lausanne). 2025 Jul 7;16:1573963. doi: 10.3389/fendo.2025.1573963 (PMC12277161; doi:10.3389/fendo.2025.1573963)
Supplement: Supplementary file 1 [file Table1.docx]

Supplementary Material

Supplementary Table 1. Multiple stepwise regression analysis of influencing factors of TITR.

| Variables | β | t | *P* | 95%CI |
| --- | --- | --- | --- | --- |
| (Constant) | 61.700 | 10.093 | <0.001 | 49.702 to 73.698 |
| HbA1c | -4.616 | -11.734 | <0.001 | -5.388 to -3.844 |
| 25(OH)D | 0.530 | 4.538 | <0.001 | 0.301 to 0.759 |
| TG | -1.255 | -4.189 | <0.001 | -1.843 to -0.667 |
| Duration | -0.522 | -4.671 | <0.001 | -0.741 to -0.303 |
| HDL-C | 7.261 | 2.348 | 0.019 | 1.191 to 13.332 |

dependent variable: TITR

Supplementary Table 2. Multiple stepwise regression analysis of influencing factors of TAR.

| Variables | β | t | *P* | 95%CI |
| --- | --- | --- | --- | --- |
| (Constant) | 19.833 | 2.318 | 0.021 | 3.039 to 36.627 |
| HbA1c | 4.992 | 13.793 | <0.001 | 4.281 to 5.702 |
| TG | 1.667 | 5.952 | <0.001 | 1.117 to 2.217 |
| Duration | 0.655 | 6.351 | <0.001 | 0.453 to 0.857 |
| 25(OH)D | -0.485 | -4.457 | <0.001 | -0.698 to -0.271 |
| HDL-C | -9.131 | -3.126 | 0.002 | -14.864 to -3.398 |
| UA | -0.022 | -2.665 | 0.008 | -0.037 to -0.006 |
| UACR | 0.002 | 2.391 | 0.017 | 0.000 to 0.005 |
| ALT | 0.069 | 2.413 | 0.016 | 0.013 to 0.125 |
| BMI | -0.433 | -1.996 | 0.046 | -0.858 to -0.007 |

dependent variable: TAR

Supplementary Table 3. The comparison of baseline characteristics by tertiles (T1-T3) of 25(OH)D levels among 582 patients undergoing a 100g standardized steamed bread meal test.

| **Variables** | **T1** | **T2** | **T3** | ***P*** |
| --- | --- | --- | --- | --- |
| number | 194 | 194 | 194 | - |
| Male n (%) | 116(59.80) | 121(62.40) | 137(70.60) | 0.067 |
| Age (years) | 56.00(46.00,65.00) | 56.00(49.75,65.00) | 55.00(48.00,64.00) | 0.489 |
| Duration (years) | 5.50(2.00,12.25) | 8.00(2.00,14.00) | 6.00(2.00,10.00) | 0.085 |
| Smoking n (%) | 39(20.10) | 43(22.20) | 51(26.30) | 0.336 |
| Drinking n (%) | 28(14.40) | 29(14.90) | 34(17.50) | 0.668 |
| Hypertension n (%) | 111(57.20) | 109(56.20) | 104(53.60) | 0.762 |
| SBP (mmHg) | 132.00(123.00,147.00) | 130.00(120.00,141.25) ^*^ | 130.00(120.00,140.00) | 0.026 |
| DBP (mmHg) | 80.00(74.00,88.00) | 78.00(73.00,87.00) | 78.00(70.00,85.00) | 0.121 |
| BMI (kg/m^2^) | 25.60(23.45,27.80) | 25.35(22.91,27.49) | 25.41(23.03,27.06) | 0.458 |
| Ca (mmol/L) | 2.21(2.13,2.27) | 2.19(2.14,2.26) | 2.20(2.13,2.27) | 0.893 |
| ALT (U/L) | 19.00(13.00,30.25) | 18.00(14.00,25.25) | 20.00(14.00,30.25) | 0.334 |
| AST (U/L) | 17.00(14.00,21.25) | 16.50(14.00,21.00) | 17.00(14.00,24.00) | 0.347 |
| TC (mmol/L) | 4.66±1.09 | 4.48±1.06 | 4.38±0.95^*^ | 0.025 |
| TG (mmol/L) | 1.84(1.16,2.60) | 1.73(1.15,2.67) | 1.37(0.97,2.03) ^*#^ | <0.001 |
| HDL-C (mmol/L) | 1.02(0.88,1.19) | 1.01(0.86,1.17) | 1.09(0.94,1.23) ^#^ | 0.003 |
| LDL-C (mmol/L) | 2.71(2.32,3.33) | 2.69(2.02,3.36) | 2.69(2.15,3.37) | 0.503 |
| Scr (mmol/L) | 56.50(45.88,67.65) | 55.20(45.53,68.43) | 58.85(50.28,69.00) | 0.121 |
| SUA (mmol/L) | 322.50(263.75,401.00) | 307.00(259.75,382.75) | 322.00(270.25,376.25) | 0.486 |
| UACR (mg/g) | 12.00(6.17,78.73) | 11.40(6.59,34.26) | 10.02(4.85,21.92) ^*^ | 0.007 |
| HbA1C (%) | 8.70(7.40,10.03) | 8.25(7.20,9.80) | 7.70(6.80,9.25) ^*^ | 0.001 |
| eGFR (ml/min per 1.73 m^2^) | 106.10(95.95,118.78) | 105.98(96.87,115.36) | 105.39(97.58,114.28) | 0.713 |
| HOMA-IR | 1.77(0.83,3.38) | 2.00(1.02,4.55) | 1.62(0.81,3.53) | 0.121 |
| HOMA-β | 26.29(13.78,64.00) | 40.93(16.00,87.56) ^*^ | 32.71(15.71,71.87) | 0.025 |
| FINS(μU/ml) | 4.95(2.48,9.82) | 6.13(3.28,13.13) | 5.55(2.47,10.30) | 0.052 |
| FCp (pmol/L) | 1.39(0.88,2.04) | 1.51(0.89,2.19) | 1.51(1.01,2.09) | 0.450 |
| FBG (mmol/L) | 7.60(6.28,8.90) | 6.85(5.80,8.80) | 6.90(5.90,8.30) ^*^ | 0.016 |
| DR n (%) | 50(25.80) | 48(24.70) | 43(22.20) | 0.694 |
| DKD n (%) | 49(25.30) | 24(12.40) ^*^ | 23(11.90) ^*^ | <0.001 |
| DPN n (%) | 54(27.80) | 55(28.40) | 39(20.10) | 0.113 |
| Anti-diabetic treatment n (%) | | | | |
| Insulin | 119(61.30) | 108(55.70) | 97(50.00) | 0.080 |
| Glucagon like peptide-1 | 35(18.00) | 36(18.60) | 31(16.00) | 0.779 |
| Metformin | 141(72.70) | 141(72.70) | 143(73.70) | 0.966 |
| Sulfonylureas | 14(7.20) | 17(8.80) | 19(9.80) | 0.660 |
| Glinides | 16(8.20) | 22(11.3) | 11(5.70) | 0.132 |
| α-glucosidase inhibitors | 73(37.60) | 62(32.00) | 73(37.60) | 0.404 |
| Thiazolidinediones | 12(6.20) | 8(4.10) | 10(5.20) | 0.656 |
| DPP-4 inhibitors | 55(28.40) | 64(33.00) | 44(22.70) | 0.077 |
| SGLT-2 inhibitors | 9(4.60) | 1(0.50) | 8(4.10) | 0.038 |
| Anti-hypertensive treatment n (%) | | | | |
| RAAS inhibitors | 84(43.30) | 69(35.60) | 71(36.60) | 0.236 |
| Calcium channel blockers | 61(31.40) | 57(29.40) | 70(36.10) | 0.352 |
| b-blockers | 22(11.30) | 21(10.80) | 24(12.40) | 0.889 |
| Diuretics | 17(8.80) | 14(7.20) | 19(9.80) | 0.660 |
| Aspirin therapy, n (%) | 41(21.10) | 35(18.00) | 33(17.00) | 0.556 |
| Statin therapy, n (%) | 72(37.10) | 65(33.50) | 66(34.00) | 0.722 |

Continuous variables are presented as means ± SD and medians (lower and upper quartiles), and categorical variables are expressed as numbers (percentages).

Abbreviations: SBP, systolic blood pressure; DBP, diastolic blood pressure; BMI, body mass index; ALT, alanine aminotransferase; AST, aspartate aminotransferase; TC, total cholesterol; TG, triglyceride; HDL-C, high-density lipoprotein cholesterol; LDL-C, low-density lipoprotein cholesterol; Scr, serum creatinine; SUA, serum uric acid; UACR, urine albumin creatinine ratio; HbA1c, Hemoglobin A1c; eGFR, estimated glomerular filtration rate; HOMA-IR, homeostatic model assessment of insulin resistance; HOMA-β, homeostasis model assessment of β cell function; FINS, Fasting insulin; FCp, Fasting C-peptide; FBG, fasting blood glucose; DR, diabetic retinopathy; DKD, diabetic kidney disease; DPN, diabetic peripheral neuropathy; DPP-4 inhibitors, Dipeptidyl Peptidase-4 inhibitors; SGLT-2 inhibitors, sodium glucose cotransporter 2 inhibitors; RAAS inhibitors, renin-angiotensin-aldosterone system inhibitors.

G1 (25 (OH)D<21.22 ng/mL), G2 (21.22 ng/mL≤25 (OH)D <25.96 ng/mL), G3 (25 (OH)D≥25.96 ng/mL).

* Significant difference with group 1 (*P* < 0.05).

# Significant difference with group 2 (*P* < 0.05).
